# Rare cause of severe anemia due to pyogenic granuloma in the jejunum

**DOI:** 10.1186/s12876-015-0355-6

**Published:** 2015-10-06

**Authors:** Shun-ichi Misawa, Hiroto Sakamoto, Akira Kurogochi, Yasushi Kirii, Shinichiro Nakamura, Tomoko Misawa, Suguru Yoneda, Mari Hirano, Osamu Owa, Hiroyuki Takagi, Hiroyoshi Ota

**Affiliations:** Department of Surgery, Matsumoto City Hospital, 4417-180 Hata, Matsumoto, Nagano 3901401 Japan; Department of Gastroenterology, Matsumoto City Hospital, 4417-180 Hata, Matsumoto, Nagano 3901401 Japan; Department of Biomedical Laboratory Sciences, Shinshu University, School of Medicine, 3-1-1 Asahi, Matsumoto, 3908621 Nagano Japan

**Keywords:** Pyogenic granuloma, Small intestine, Double-balloon endoscopy, Obscure gastrointestinal bleeding, Hypervascular tumor

## Abstract

**Background:**

Pyogenic granuloma (PG) is a polypoid lobular capillary hemangioma rarely observed in the gastrointestinal tract. Only a few cases in the small bowel have been described in the literature.

**Case presentation:**

A 58-year-old man had been suffering from general fatigue and severe anemia. Esophagogastroduodenoscopy and colonoscopy did not reveal any significant bleeding. Abdominal computer tomography revealed a hypervascular tumor in the small intestine. Oral double-balloon endoscopy (DBE) detected a polypoid lesion (2 cm in diameter) in the jejunum. We performed laparoscopic-assisted partial resection of the jejunum. The histological features of the tumor were consistent with PG. The patient’s anemia gradually improved without the need for oral iron after surgery.

**Conclusion:**

In this case report, we present a case of pyogenic granuloma in in the jejunum that was detected by DBE.

## Background

Obscure gastrointestinal bleeding (OGIB) is frequently observed in the small intestine. Double-balloon endoscopy (DBE) and capsule endoscopy (CE) are highly effective in diagnosing the origin of OGIB [1, 2]. DBE is particularly valuable for the detection and diagnosis of small bowel tumors, and biopsies and therapeutic procedures have also become possible [3].

Pyogenic granuloma (PG) is a capillary hemangioma that usually occurs on the skin or in the oral cavity; it is rarely observed in the gastrointestinal tract. We report a case of PG in the jejunum that was detected by DBE.

## Case presentation

**Fig. 1:**
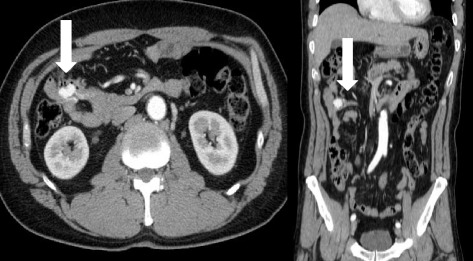
Abdominal enhanced computed tomography showing a 2-cm hypervascular tumor in the small intestine

**Fig. 2:**
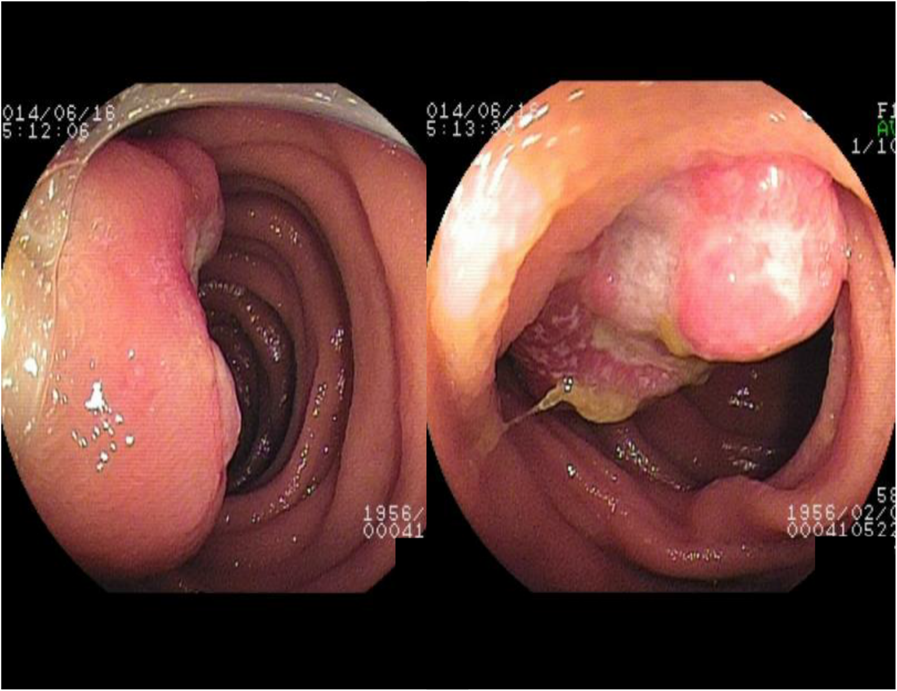
Double-balloon endoscopy findings of a submucosal tumor with surface ulceration in the jejunum

A 58-year-old man had been suffering from general fatigue and severe anemia for several months. His hemoglobin levels were 6.6 g/dl (normal range: 12–16 g/dl). He had no medical history and did not take any medicine. Esophagogastroduodenoscopy and colonoscopy did not reveal any significant bleeding. Abdominal computer tomography revealed a 2-cm hypervascular tumor in the small intestine (Fig. 1). Oral DBE detected a 2-cm-diameter reddish, submucosal tumor-like lesion with surface ulceration in the jejunum, approximately 20 cm away from the Treitz ligament (Fig. 2). We did not perform biopsy because it can be difficult to stop bleeding in the case of hypervascular lesions. Under the diagnosis of a small bowel tumor, gastrointestinal stromal tumor (GIST), malignant lymphoma, or cancer, we performed laparoscopic-assisted segmental resection of the jejunum with the dissection of lymph nodes.

**Fig. 3:**
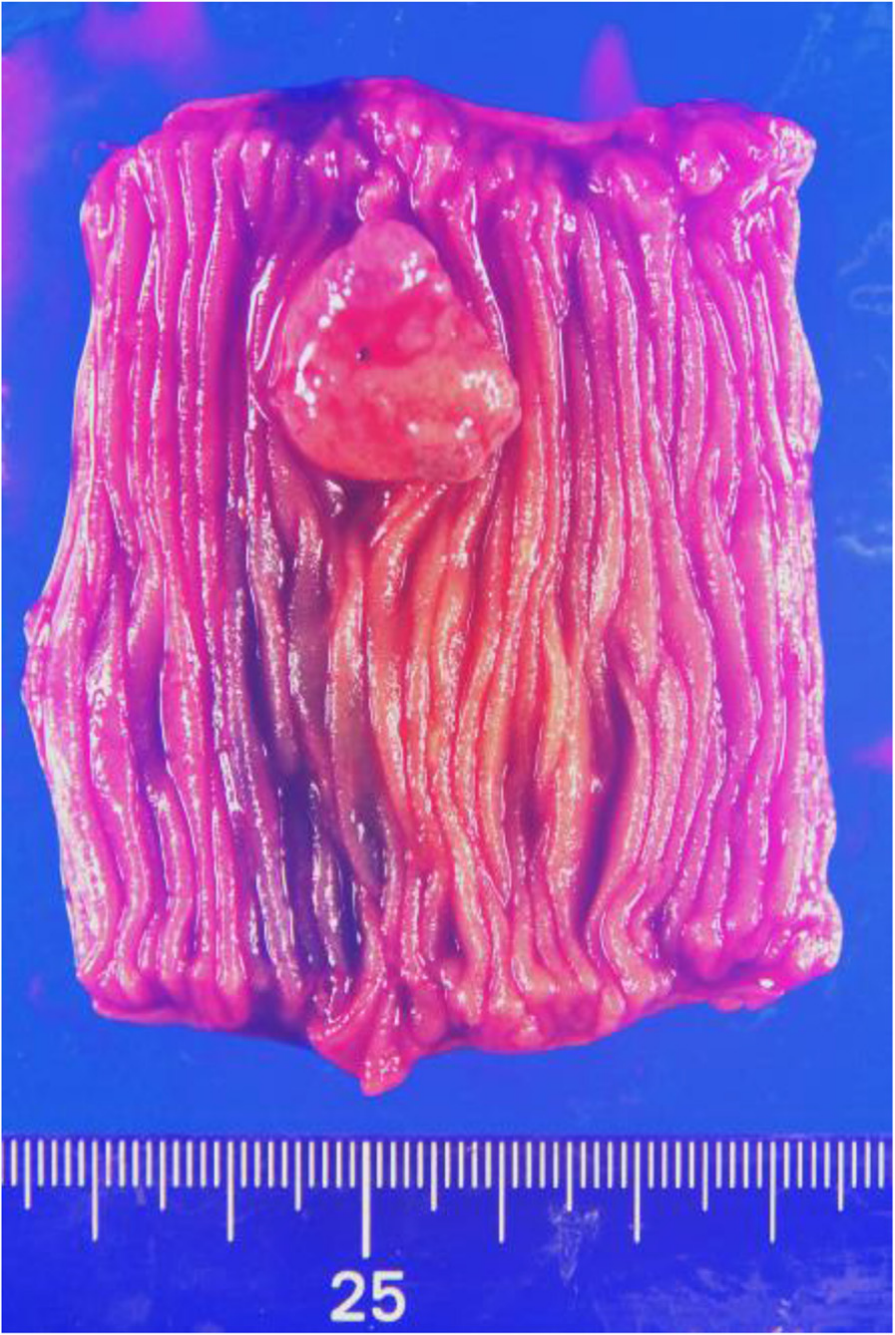
Macroscopic appearance of the resected tumor showing a 19 × 16 mm subpedunculated polyp with surface ulceration

**Fig. 4:**
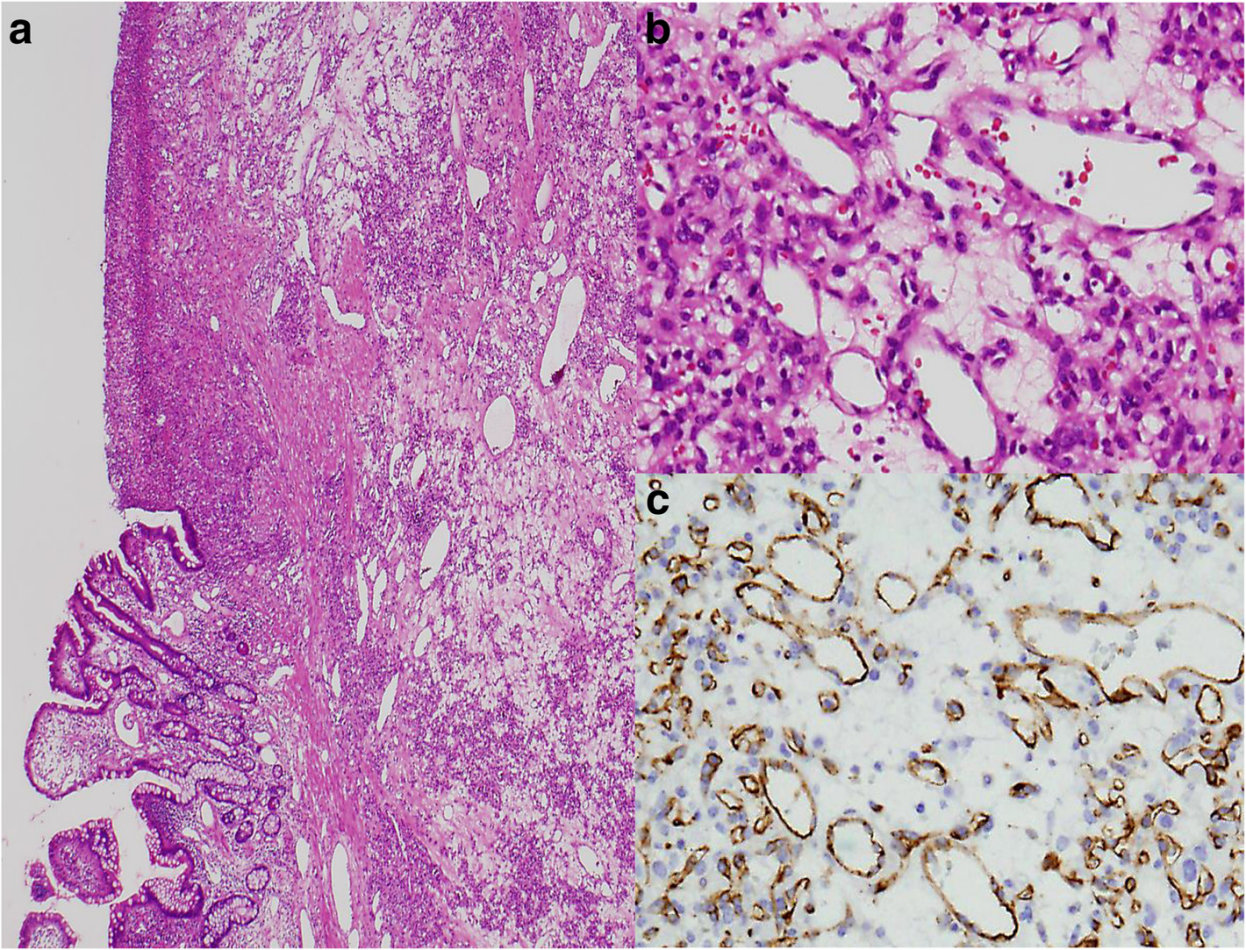
Microscopic appearance of the hematoxylin and eosin (H&E) stained resected specimen. **a** Low-power view of the histological features. **b** High-power view of the histological features (original magnification: ×100). Proliferation and lobular arrangement of capillary-sized blood vessels can be observed along with inflamed and edematous stroma and endothelial cell swelling. Remarkable inflammatory cell invasion and granulation tissues are noted in the capillaries and granulation tissue. **c** Positive immunostaining for CD31 in capillary endothelial cells

Examination of the resected tumor showed that it measured 19 × 16 mm in diameter (Fig. 3). Histology revealed the proliferation of blood capillaries and granulation tissue, which was consistent with PG (Fig. 4). The patient was discharged on postoperative day 9 without complication and his anemia improved gradually without the need for oral iron after surgery.

## Discussion

PG, also called granuloma telangiectaticum or lobular capillary hemangioma, was first described in 1897. Gastrointestinal PG is apparently a rare cause of hemorrhage in the digestive tract. PG is a benign lesion that is considered reactive, e.g., it occurs as a result of minor trauma. However, the precise etiology is unknown. Satellite lesions have been described on the skin, which argues against trauma as the sole cause. Lesions are also found more frequently during pregnancy on the skin and in the mouth, suggesting that hormonal influences may also play a role. All gastrointestinal PGs reported thus far have occurred in elderly patients, both male and female [4]. The distinctive histopathological characteristics of PGs are proliferation and lobular arrangement of capillary-sized vessels with inflamed and edematous stroma and endothelial cell swelling cells [5].

To our knowledge, only 16 cases of PG in the small intestine have been described in the literature, and most involved Japanese patients (Table 1). This may be due to the development of DBE for examination of the small bowel in Japan. Including our case, the average age of the 17 patients was 58.1 years (range: 26–86 years), with no significant difference between males and females.

**Table 1:** Summary of the reported cases of PG in the small intestine

Author	Year	Age/Sex	Symptoms	Hb (g/dl)	Location	Size (mm)	Treatment
Payson [7]	1967	45/F	Abdominal pain	12.4	Ileum	60	Surgical resection
Meuwissen [8]	1986	37/M	None	NA	Ileum	NA	Laser ablation
Iwakubo [9]	1989	30/F	Melena	4.8	Ileum	8 × 8	Surgical resection
Hizawa [10]	1993	26/F	None	NA	Ileum	NA	Surgical resection
Yao [11]	1995	71/F	Anemia	NA	Ileum	24	Surgical resection
Yao [11]	1995	56/M	Melena	NA	Jejunum	20	Surgical resection
Motohashi [12]	1999	58/F	Anemia	6.1	Ileum	25 × 30	Surgical resection
Van Eden [4]	2004	55/F	Anemia	NA	Ileum	9	Surgical resection
Shirakawa [13]	2007	72/M	Anemia	NA	Ileum	NA	EMR
CHO [6]	2009	58/M	Melena	5	Ileum	10	Surgical resection
Moffat [14]	2009	78/M	Anemia	6.2	Jejunum	20	Surgical resection
Nagoya [5]	2009	63/F	Anemia	4	Ileum	7	EMR
Kuga [15]	2009	55/M	Anemia	9.9	Jejunum	4	EMR
Yamashita [16]	2013	61/M	Anemia	NA	Ileum	15	Surgical resection
Katsurahara [17]	2015	65/M	Anemia	NA	Jejunum	33	Surgical resection
Kikuchi [18]	2015	86/F	Anemia	8.1	Ileum	7	Surgical resection
This case	2015	58/M	Anemia	6.6	Jejunum	19	Surgical resection

*NA* data not available

The tumor size is usually less than 20 mm, with a clinical presentation of anemia. Of the 17 reported cases, 12 involved the ileum and 5 involved the jejunum. Most tumors had an irregular shape without surface ulceration and were reddish in color. Surgical resection is the mainstay for treating small bowel PGs, although endoscopic resection with an electrosurgical snare is an alternative technique if an endoscopic approach is possible. Endoscopic treatment is a relatively easy, safe, and low-cost procedure for small-sized PGs of the gastrointestinal tract [3]. However, when resecting PGs, it is important that arteriovenous anastomosis under the tumor is included endoscopically, because incomplete resection may cause recurrence [6].

In the present case, we performed laparoscopic-assisted partial resection because complete endoscopic resection of the 2-cm hypervascular submucosal tumor with ulceration seemed to be difficult.

## Conclusion

PG in the small intestine is a rare cause of anemia and is difficult to detect. Awareness regarding this infrequent benign lesion will make it easier to diagnose and treat it properly.

## Consent

Written informed consent was obtained from the patient for publication of this case report and any accompanying images.

## Abbreviations

PG: Pyogenic granuloma
OGIB: Obscure gastrointestinal bleeding
DBE: Double-balloon endoscopy
CE: Capsule endoscopy
GIST: Gastrointestinal stromal tumor
